# Oxidation Prevents HMGB1 Inhibition on PDGF-Induced Differentiation of Multipotent Vascular Stem Cells to Smooth Muscle Cells: A Possible Mechanism Linking Oxidative Stress to Atherosclerosis

**DOI:** 10.1155/2018/4019814

**Published:** 2018-05-23

**Authors:** Xiaohu Meng, Wenjie Su, Xuan Tao, Mingyang Sun, Rongchao Ying, Wei Wei, Baolin Wang

**Affiliations:** ^1^Division of Vascular Surgery, The Second Affiliated Hospital of Nanjing Medical University, Nanjing, China; ^2^Department of General Surgery, Hangzhou First People's Hospital Affiliated to Nanjing Medical University, Hangzhou, China

## Abstract

Atherosclerosis is considered as a multifactorial disease in terms of the pathogenic mechanisms. Oxidative stress has been implicated in atherogenesis, and the putative mechanisms of its action include oxidative modification of redox-sensitive signaling factors. High mobility group box 1 (HMGB1) is a key inflammatory mediator in atherosclerosis, but if oxidized it loses its activity. Thus, whether and how it participates in oxidative stress-induced atherosclerosis are not clear. The current study found that exogenous HMGB1 dose-dependently inhibited the proliferation of multipotent vascular stem cells and their differentiation to smooth muscle cells induced by platelet-derived growth factor. But oxidative modification impaired the activity of HMGB1 to produce the effect. The stem cells were regarded as the source of smooth muscle cells in vascular remodeling and neointimal hyperplasia. Therefore, the findings suggested that HMGB1 participated in oxidative stress-induced atherosclerosis presumably by targeting multipotent vascular stem cells.

## 1. Introduction

Atherosclerosis is a leading cause of heart attack, stroke, and peripheral vascular disease. It is characterized by chronic obliterative vasculopathy resulting from subendothelial accumulation of abundant fatty substance, macrophages, and smooth muscle cells (SMCs) creating an atheromatous plaque [1]. Although the buildup of plaque is a slow process in response to a variety of local vascular factors, oxidative stress has been linked to atherogenesis [2]. Oxidative stress displays a high level of reactive oxygen species (ROS) that is mainly caused by an imbalance between the activity of endogenous prooxidative and antioxidative enzymes in favor of the former. In vascular walls, many ROS-producing enzyme systems are present, consisting of nicotinamide adenine dinucleotide phosphate oxidase, xanthine oxidase, enzymes of mitochondrial electron transport chain, and uncoupled endothelial nitric oxide synthase. ROS function as important intercellular second messengers to modulate many downstream signaling molecules, such as transcription factors, mitogen-activated protein kinases, and ion channels. Induction of these signaling cascades results in the expression of proinflammatory genes, SMC growth, and migration [2, 3]. Excessive ROS production can also damage essentially all biomolecules, including DNA, protein, and lipids, which consequently aggravates vascular injury [3]. However, the molecular and cellular events that are involved in atherogenesis driven by oxidative stress remain elusive and need further investigation, among which much effort is given to explore redox-sensitive signaling factors. High mobility group box 1 (HMGB1) is an intriguing molecule which has at least three redox forms with different biological functions [4]. The protein contains three conserved redox-sensitive cysteine residues: C23 and C45 can form an intramolecular disulfide bond, whereas C106 is unpaired. Many of the intracellular and extracellular functions of HMGB1 depend on redox-sensitive cysteine residues of the protein [5]. Intercellular HMGB1 with all three cysteines fully reduced, namely, all-thiol HMGB1, can translocate to the nucleus to bind to DNA and regulate gene expression, which is inhibited by oxidation [6, 7]. Extracellular HMGB1 is increased at atherosclerotic plagues by passive release from necrotic cells and secretion by inflammatory cells [4]. It exists in both all-thiol and disulfide-bonded forms, acting as a potent inflammatory cytokine to mediate the activation of innate immune responses, including chemotaxis and cytokine release. All-thiol HMGB1 exerts the chemotactic activity to recruit inflammatory cells by forming a heterocomplex with CXCL12 and signaling exclusively via CXCR4 [8], whereas disulfide HMGB1 binds and signals via the TLR4/MD-2 complex to induce cytokine release by macrophages [9]. Moreover, HMGB1 stimulates SMC proliferation and migration to form atheromatous plaques [4]. However, the bioactivity of HMGB1 is abrogated by oxidation of all cysteine residues when inflammation resolves [5]. Therefore, it is not known whether and how HMGB1 are engaged in oxidative stress-induced vascular injury due to its property of oxidative inactivation. The present study proposed that HMGB1 participated in oxidative stress-induced atherosclerosis by targeting multipotent vascular stem cells (MVSCs). MVSCs are a specific group of stem cells that exist physiologically as a very small population in the tunica media of mature blood vessels and express markers including Sox17, Sox10, S100*β*, and neural filament-medium polypeptide (NFM), but not smooth muscle myosin heavy chain (SM-MHC) [10]. The stem cells had not been identified until recently, when they were successfully isolated and found to have the capacity of SMC differentiation [10]. This study suggested that the activation and differentiation of MVSCs, but not the dedifferentiation of mature contractile SMCs, led to the generation of proliferative and synthetic SMCs in the vasculature, which contributed to vascular lesion formation [10]. Moreover, oxidative stress was revealed to regulate MVSCs differentiation, since nitric oxide which possesses the activity of inhibiting oxidative stress suppressed their differentiation to mesenchymal stem cell-like cells and subsequently to SMCs [11]. The current study found that oxidative modification impaired HMGB1 inhibition on the proliferation of MVSCs and their differentiation to SMCs induced by platelet-derived growth factor (PDGF), which presumably resulted in MVSCs activation and their engagement in atherogenic process. Therefore, we postulated a novel mechanism linking oxidative stress to atherosclerosis, which considered HMGB1 as a critical regulator in oxidative stress-induced vascular injury.

## 2. Material and Methods

### 2.1. Animals

F344 rats were purchased from Vital River (Beijing, China) and received humane care in compliance with the National Institutes of Health Guide for the Care and Use of Laboratory Animals (NIH Publications No. 8023, revised 1978). The procurement of rat aorta for study use was approved by the Committee of Animal Experiment Ethnics at Nanjing Medical University.

### 2.2. Chemicals and Reagents

Cell culture media and supplements were procured as follows: Dulbecco's modified Eagle medium (DMEM, Invitrogen), fetal bovine serum (FBS, Invitrogen), chick embryo extract (MP Biomedical), N2 (Invitrogen), B27 (Invitrogen), basic fibroblast growth factor (R&D Systems), retinoic acid (Sigma-Aldrich), 2-mercaptoethanol (Sigma-Aldrich), penicillin/streptomycin (Invitrogen), and PDGF-BB (ProSpec). Antibodies were obtained from Abcam and listed below: HMGB1 antibody (HMGB1 Ab), SM-MHC antibody, Sox17 antibody, Sox10 antibody, NFM antibody, S100*β* antibody, tubulin antibody, Alexa Fluor 488 conjugated goat anti-mouse antibody, Alexa Fluor 488 conjugated goat anti-rabbit antibody, and Alexa Fluor 647 conjugated goat anti-rabbit antibody. Other reagents together with their manufactures were listed as follows: recombinant HMGB1 protein (Sigma-Aldrich), CCK-8 kit (Beyotime Biotech), and CBD Cytofix/Cytoperm solution (BD Bioscience). Oxidized HMGB1 was prepared by using hydrogen peroxide and detected by Western blot as previously described [12]. Briefly, recombinant HMGB1 was pretreated with hydrogen peroxide at a gradient concentration of 5, 16.7, and 50 *μ*M (*μ*mol/l) to generate a series of mixtures of inoxidized and oxidized HMGB1. The fraction of oxidized HMGB1 was separated by Western blot and calculated from band intensity of oxidized HMGB1 that was normalized to the total of inoxidized and oxidized HMGB1. Consequently, the mixtures with approximately 30% and 50% oxidized HMGB1 were obtained upon 16.7 *μ*M and 50 *μ*M hydrogen peroxide treatment. They were selected for the subsequent experiments and labeled as LO-HMGB1 (low-level oxidized HMGB1) and HO-HMGB1 (high-level oxidized HMGB1), respectively. Hydrogen peroxide at 5 *μ*M had little effect on HMGB1 oxidation (Figure S1).

### 2.3. MVSC Isolation and Culture

Rat MVSCs were isolated from abdominal aorta with the tissue explant method as described previously [10]. Briefly, F344 rat aorta was procured and dissected in a sterile environment. The tunica media were separated from the adjacent adventitia and endothelium, sectioned into 2 mm cubes, and placed into a 6-well plate coated with Cellstart to allow their attachment. The growth medium (DMEM supplemented with 10% FBS) was added at 2 mL per well to stimulate cell growth at 37°C and 5% CO_2_ until it was replaced by an equal volume of maintenance medium two weeks later to prevent cell differentiation. The maintenance medium was prepared from DMEM mixed with 2% chick embryo extract, 1% FBS, 1% N2, 2% B27, 100 nM retinoic acid, 50 nM 2-mercaptoethanol, 1% penicillin/streptomycin, and 20 ng/mL basic fibroblast growth factor. The tissue explants were removed timely if detached or kept in culture medium until the end of two weeks. When the cells were grown to 80% confluence, they were transferred to the flasks for culture expansion.

### 2.4. Proliferation Assay

Cell proliferation was measured by CCK-8 assay. Briefly, MVSCs at passage 2 were seeded in a 96-well plate at a density of 4 × 10^3^ cells per well and treated for 24 h with HMGB1, HMGB1 + HMGB1 Ab (diluted to 1 : 1000), and oxidized HMGB1, respectively. Then the cells were incubated in fresh medium containing 10% CCK8 reagent for 1-2 h in the dark. The absorbance at 450 nm for each well was measured by a microplate reader. The experiments were repeated five times for each group to calculate the average absorbance values which were subsequently normalized to that of untreated cells to represent the cell survival.

### 2.5. Transwell Assay

Cell migration was evaluated by transwell assay. Firstly, transwell inserts with 8 *μ*m pore membrane filters (Corning) were inserted into the chambers of 24-well plates which were consequently partitioned into the upper and lower compartments. Next, 8 × 10^4^ cells were seeded in each upper compartment, and the lower compartment contained HMGB1, HMGB1 + HMGB1 Ab, and oxidized HMGB1, respectively. After being cultured for 24 h, the cells that migrated to the lower side of membrane were fixed in methanol, stained with 0.1% crystal violet solution, and visualized by phase-contrast microscopy. The experiments were repeated five times for each group, and data were expressed as the average migrated cell count per ×200 field micrograph.

### 2.6. Cell Differentiation

The differentiation of MVSCs to SMCs was stimulated by PDGF-BB as previously described [10]. Briefly, the isolated MVSCs at passage 2 were cultured in DMEM supplemented with 5% FBS and 50 ng/ml PDGF-BB for 7 days. The expression of SM-MHC was detected by immunofluorescence and flow cytometry to determine the population of differentiated cells.

### 2.7. Cell Immunofluorescence

The cells were fixed with 4% paraformaldehyde, permeabilized with 0.5% Triton X-100, and blocked by 5% normal goat serum. Target protein was probed with specific primary antibodies and then visualized by incubation with corresponding fluorescence-labeled secondary antibodies. Finally, the nuclei were counterstained with 4′,6-diamidino-2-phenylindole (DAPI).

### 2.8. Flow Cytometry

Cell samples were dissociated by Trypsin/EDTA solution, fixed, and permeabilized by BD Cytofix/Cytoperm solution. Each sample was evenly divided into two parts which were incubated with specific primary antibody and its isotype antibody, respectively, followed by secondary antibody binding. Flow cytometric analyses were performed by using a BD FACSCanto II flow cytometer (BD Biosciences). Three independent experiments were performed for each analysis.

### 2.9. Statistical Analysis

Data analysis was performed using GraphPad Prism 5. Numerical data were expressed as mean ± standard deviation and compared between groups by one-way ANOVA. A *P* value < 0.05 indicated significant difference.

## 3. Results and Discussion

**Figure 1:**
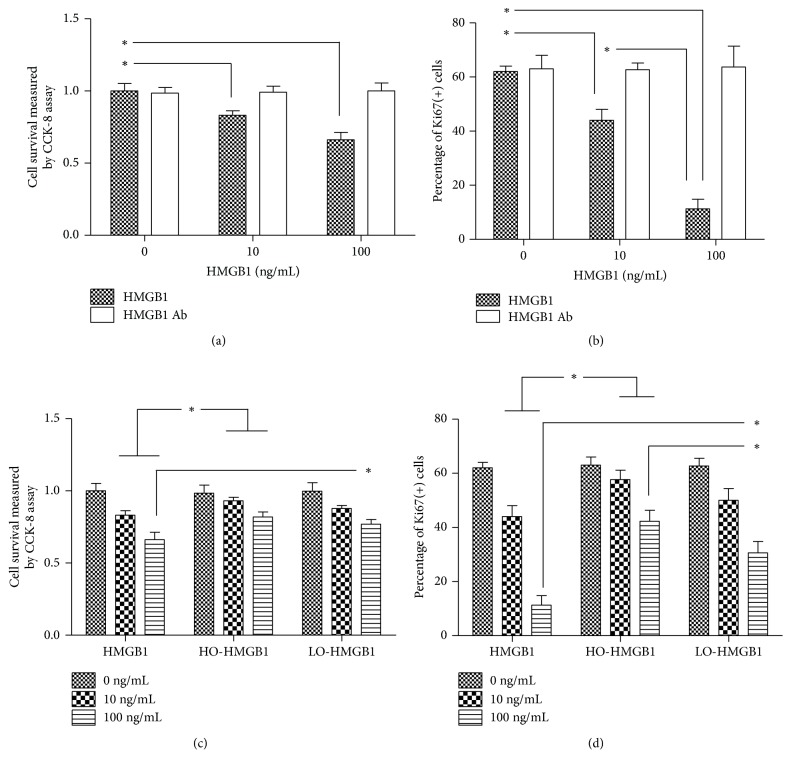
The assessment of MVSC proliferative capacity after HMGB1 and oxidized HMGB1 treatment. The cells at passage 2 were treated with HMGB1, LO-HMGB1, and HO-HMGB1 at the concentration of 10 ng/ml and 100 ng/ml for 24 h. The negative control was HMGB1-untreated cells (the groups at 0 ng/ml). In CCK8 assay, cell survival was represented by average absorbance at 450 nm that was calculated from five repeated experiments and normalized to that of untreated cells. (a) HMGB1 treatment inhibited cell survival in a dose-dependent manner, which was blocked by HMGB1 Ab. (b) HO-HMGB1 at both 10 ng/ml and 100 ng/ml had less effect than HMGB1 on cell growth inhibition. The difference in cell growth between LO-HMGB1 and HMGB1 treatment groups was significant only at 100 ng/ml. In flow cytometry, the average percentage of Ki67-positive cells was calculated from three repeated tests. (c) HMGB1 treatment decreased the percentage of Ki67-positive cells, and HMGB1 Ab blocked the effect. (d) The percentage of Ki67-positive cells was significantly increased by 10 ng/ml and 100 ng/ml HO-HMGB1 treatment as compared to HMGB1 treatment. The difference in percentage of Ki67-positive cells between LO-HMGB1 and HMGB1 treatment was significant only at 100 ng/ml. Similarly, the percentage of Ki67-positive cells between LO- and HO-HMGB1 treatment groups was not significantly different until at 100 ng/ml. Group comparisons were made using one-way ANOVA. ^*∗*^*P* < 0.05.

**Figure 2:**
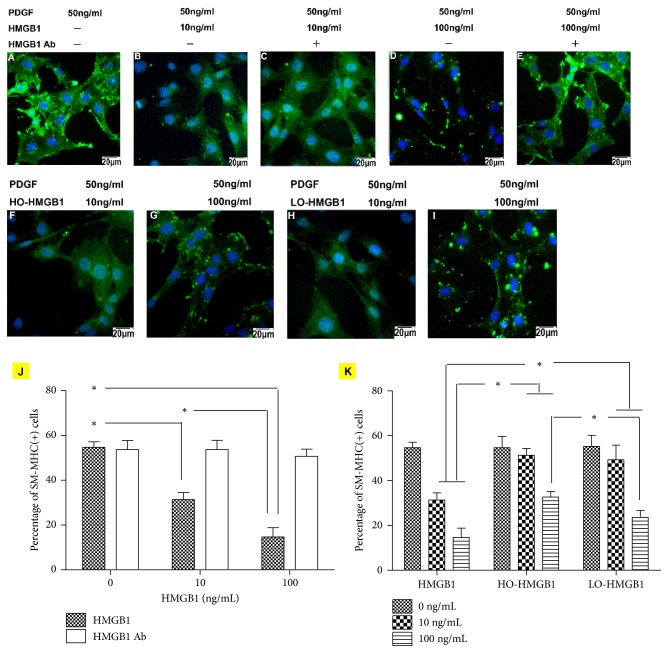
The effect of HMGB1 and oxidized HMGB1 treatment on PDGF-induced differentiation of MVSCs to SMCs. The MVSCs were treated with PDGF-BB for 7 days to induce SMC differentiation. SM-MHC was detected by immunofluorescent assay to identify differentiated cells. HMGB1 treatment reduced the population of SM-MHC-positive cells (A, B, and D), which was blocked by HMGB1 Ab (C, E). In contrast, HO- and LO-HMGB1 treatment had less effect on the capacity of cell differentiation to SMCs (F–I). The results were confirmed by flow cytometry for SM-MHC-positive cells (J, K). HMGB1 treatment reduced the percentage of SM-MHC-positive cells in total cell population, and HMGB1 Ab blocked the effect (J). The percentages of SM-MHC-positive cells were significantly higher in HO- and LO-HMGB1 treatment groups than HMGB1 treatment group (K). The percentage of SM-MHC-positive cells between LO- and HO-HMGB1 treatment groups was not significantly different until at 100 ng/ml. Group comparisons were made using one-way ANOVA. ^*∗*^*P* < 0.05.

**Figure 3:**
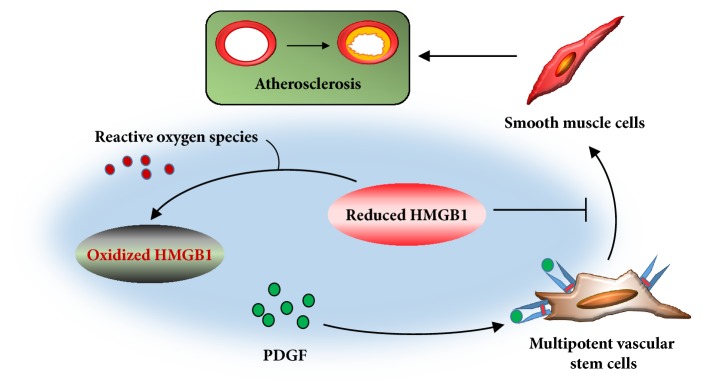
Illustration of a possible mechanism that HMGB1 mediates oxidative stress-induced atherosclerosis. Multipotent vascular stem cells proliferate and differentiate to smooth muscle cells in response to stimulatory factors (such as PDGF), which consequently accelerates atherosclerosis. HMGB1 is able to inhibit the proliferation and differentiation of multipotent vascular stem cells, but if oxidized, it loses its activity. HMGB1 oxidation is promoted by a great amount of reactive oxygen species that are generated during oxidative stress. Thus, this suggests a possible mechanism linking oxidative stress to atherosclerosis.

The isolated cells were sent to immunofluorescence assay for MVSC markers at first. The cells expressed Sox17, Sox10, S100*β*, and NFM but not SM-MHC (Figure S2). This was consistent with what was described in the previous studies and demonstrated that the isolated cells were MVSCs. Next, the cells were treated with recombinant HMGB1 at dosages of 10 ng/ml and 100 ng/ml. In the CCK-8 assay, cell survival was significantly lower than that without HMGB1 treatment, suggesting HMGB1 inhibited MVSC proliferation. The inhibitory effect was dose-dependent and blocked by HMGB1 Ab (Figure 1(a)). However, oxidation impaired HMGB1 inhibition on cell growth. The cell survival was significantly increased after HO-HMGB1 treatment in comparison with the cells treated with HMGB1 of the same concentration (Figure 1(c)). The significant discrepancy between LO-HMGB1 and HMGB1 treatment was only observed at 100 ng/ml. To verify the results, we performed flow cytometry for cellular expression of Ki67, which was an excellent marker to determine the growth fraction of MVSC population [10]. HMGB1 treatment inhibited the percentage of Ki67-positive cells in a dose-dependent manner. Ki67-positive cells were dramatically decreased when HMGB1 concentration increased from 10 ng/ml to 100 ng/ml (Figure 1(b)). But HO-HMGB1 was less effective than HMGB1 in reducing Ki67-positive cells (Figure 1(d)). Such difference was also observed between LO-HMGB1 and HMGB1 at 100 ng/ml. Thus, HMGB1 inhibition on MVSC proliferation was regulated by its redox state: oxidation impaired HMGB1 activity and subsequently promoted MVSC growth. Moreover, the present study demonstrated that oxidation attenuated HMGB1 inhibition on PDGF-induced differentiation of MVSCs to SMCs. PDGF was well established as a potent growth factor to stimulate SMC differentiation of stem cells. Approximately 60% of MVSCs differentiated to SM-MHC-positive cells after PDGF-BB treatment for 7 days. HMGB1 treatment significantly decreased the population of differentiated cells in a dose-dependent manner, and only 16% of cells differentiated at 100 ng/ml HMGB1 (Figures 2(A), 2(B), 2(D), and 2(J)). HMGB1 Ab blocked the effect of HMGB1 and restored cell differentiation capacity (Figures 2(C), 2(E), and 2(J)). This indicated that HMGB1 inhibited PDGF-induced differentiation of MVSCs to SMCs. However, if HMGB1 was oxidized prior to use, cell differentiation capacity was improved. The differentiation rates were increased to 51% and 31% at 10 ng/ml and 100 ng/ml HO-HMGB1 and 49% and 23% at 10 ng/ml and 100 ng/ml LO-HMGB1, respectively (Figures 2(F)–2(I) and 2(K)). Moreover, the difference in differentiation rates between HO- and LO-HMGB1 treatment was significant at the high concentration (100 ng/ml) and correlated with approximately a half-fold greater fraction of oxidized HMGB1 in HO-HMGB1 (Figures 2(G), 2(I), and 2(K)). Taken together, oxidation protected MVSCs from HMGB1 inhibition and subsequently promoted cell proliferation and SMC differentiation. This suggested a MVSC-targeted mechanism by which HMGB1 participated in oxidative stress-induced atherosclerosis. Recent studies suggested that the SMCs in atheromatous plaque were derived from medial MVSCs which proliferated and differentiated to SMCs in response to vascular injury [10]. HMGB1 suppressed MVSC proliferation and differentiation. But oxidative stress generated a great amount of ROS so that HMGB1 was easily oxidized and became inactive. Therefore, it was very likely that oxidative stress stimulated MVSCs by inhibition of HMGB1 to accelerate formation of atheromatous plaques (Figure 3).

**Figure 4:**
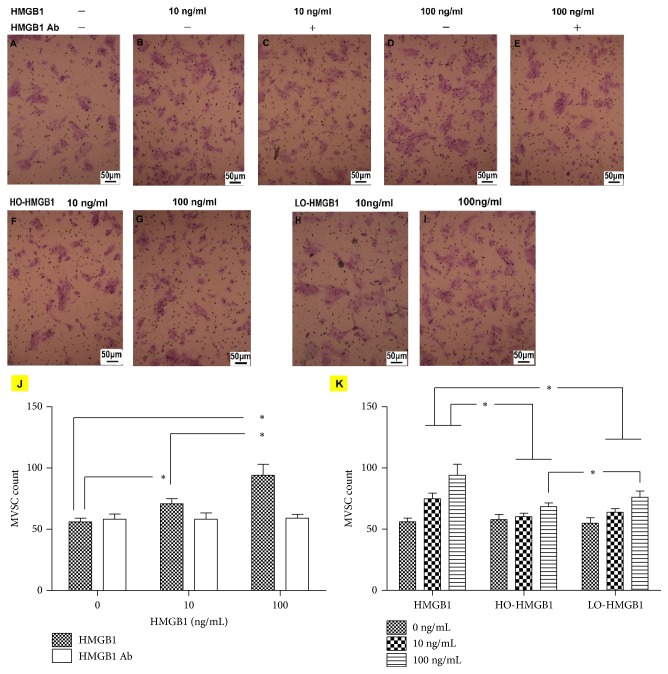
The effects of HMGB1 and oxidized HMGB1 treatment on MVSC migration. In transwell assay, 8 × 10^4^ cells in the upper chamber were allowed to migrate towards HMGB1 and oxidized HMGB1 in the lower chamber at 37°C for 24 h. The migrated cells were significantly increased by HMGB1 (A, B, D, and J), which was blocked by HMGB1 Ab (C, E). In contrast, HO- and LO-HMGB1 had less effect on cell migration (F–I, K). The difference in number of migrated cells between LO-HMGB1 and HMGB1 treatment was significant only at 100 ng/ml. Group comparisons were made using one-way ANOVA. ^*∗*^*P* < 0.05.

Our theory would be more convincing if the following points were taken into account. First, since HMGB1 suppressed MVSC activity, did it indicate that HMGB1 protected against atherosclerosis by reducing SMCs in atheromatous plaques? Obviously the answer is no. Otherwise, it was contrary to many previous studies that regarded HMGB1 as a key inflammatory mediator in atherosclerosis [4, 13]. Actually, atherosclerosis is a slow process starting from fatty streak to atheroma which displays subendothelial lipid accumulation and inflammation and later to fibroatheroma during which SMCs and extracellular matrix increase in plaques [1]. Therefore, HMGB1 is presumably instrumental in aggravating inflammation while inhibiting MVSC proliferation and differentiation in early stage of atherosclerosis. Later, inflammatory cells release ROS to make it oxidized and inactivated, leading to restoration of MVSCs capacity so that they can proliferate and differentiate to SMCs in plaques. This could also explain why HMGB1 was supposed to mediate oxidative stress-induced atherosclerosis even though oxidation was found to inhibit HMGB1-induced MVSC migration (Figure 4). The transwell assay showed that HMGB1 treatment significantly promoted MVSC migration in a dose-dependent manner, which was blocked by HMGB1 Ab (Figures 4(A)–4(E) and 4(J)). Oxidation attenuated the stimulatory effect of HMGB1 (Figures 4(F)–4(I) and 4(K)). However, the findings were not definitely contrary to our theory. In early atherosclerosis, HMGB1 probably attracts MVSCs to plaques, where they neither proliferate nor differentiate until HMGB1 is later oxidized by ROS. Finally, the differentiation of MVSCs was reported to be suppressed by ROS inhibitor [11], and our study agreed with it and provided further evidence that oxidative stress presumably promoted their differentiation by inhibition of HMGB1. However, a recent study had seemingly contradictory findings that intracellular ROS acted as a negative regulator of SMC differentiation that enabled MVSCs to remain in an undifferentiated state [14]. Admittedly, ROS at a lower and controlled level are salutary to cell growth and stemness maintenance. But when ROS accumulate indiscriminately within cells, like during atherogenesis, they can be toxic, leading to oxidative stress and possible cell death [3, 15]. Under the circumstance, a great amount of ROS released from inflammatory cells and/or necrotic cells may render biomolecules oxidized, presumably including oxidation of HMGB1. However, the phenomena are generally not reproducible in a well-controlled oxidative stress as investigated by the previous study [14].

In conclusion, the present study revealed that oxidation promoted the proliferation of MVSCs and their differentiation to SMCs induced by PDGF by inhibition of HMGB1 activity. We postulated that this was a possible mechanism linking oxidative stress to atherosclerosis, which should be justified by further investigation in animal models and clinical settings.

## Abbreviations

DAPI: 4′,6-Diamidino-2-phenylindole
DMEM: Dulbecco's modified Eagle medium
FBS: Fetal bovine serum
HMGB1: High mobility group box 1
HMGB1 Ab: HMGB1 antibody
MVSC: Multipotent vascular stem cell
NFM: Neural filament-medium polypeptide
PDGF: Platelet-derived growth factor
ROS: Reactive oxygen species
SMC: Smooth muscle cell
SM-MHC: Smooth muscle myosin heavy chain.

## Supplementary Materials

Supplementary Materials Figure S1: determination of oxidized HMGB1 content in the mixtures of inoxidized and oxidized HMGB1. Oxidized HMGB1 was separated on SDS-PAGE gel and detected by Western blot with HMGB1 antibody (A). The mixtures with approximately 30% and 50% oxidized HMGB1 were labeled as LO-HMGB1 (low-level oxidized HMGB1) and HO-HMGB1 (high-level oxidized HMGB1), respectively (B). Figure S2: immunofluorescent assay for MVSC markers on isolated cells. The cells were positive for Sox17, Sox10, neural filament-medium (NFM) polypeptide, and S100*β* while lacking smooth muscle myosin heavy chain (SM-MHC). Ki67 expression indicated that the cells were highly proliferative.
