# Supplementary material for: Oxidation Prevents HMGB1 Inhibition on PDGF-Induced Differentiation of Multipotent Vascular Stem Cells to Smooth Muscle Cells: A Possible Mechanism Linking Oxidative Stress to Atherosclerosis
Source: Biomed Res Int. 2018 May 23;2018:4019814. doi: 10.1155/2018/4019814 (PMC5989286; doi:10.1155/2018/4019814)
Supplement: Supplementary Materials — Figure S1: determination of oxidized HMGB1 content in the mixtures of inoxidized and oxidized HMGB1. Oxidized HMGB1 was separated on SDS-PAGE gel and detected by Western blot with HMGB1 antibody (A). The mixtures with approximately 30% and 50% oxidized HMGB1 were labeled as LO-HMGB1 (low-level oxidized HMGB1) and HO-HMGB1 (high-level oxidized HMGB1), respectively (B). Figure S2: immunofluorescent assay for MVSC markers on isolated cells. The cells were positive for Sox17, Sox10, neural filament-medium (NFM) polypeptide, and S100β while lacking smooth muscle myosin heavy chain (SM-MHC). Ki67 expression indicated that the cells were highly proliferative. [file 4019814.f1.pdf]

Supplementary Material

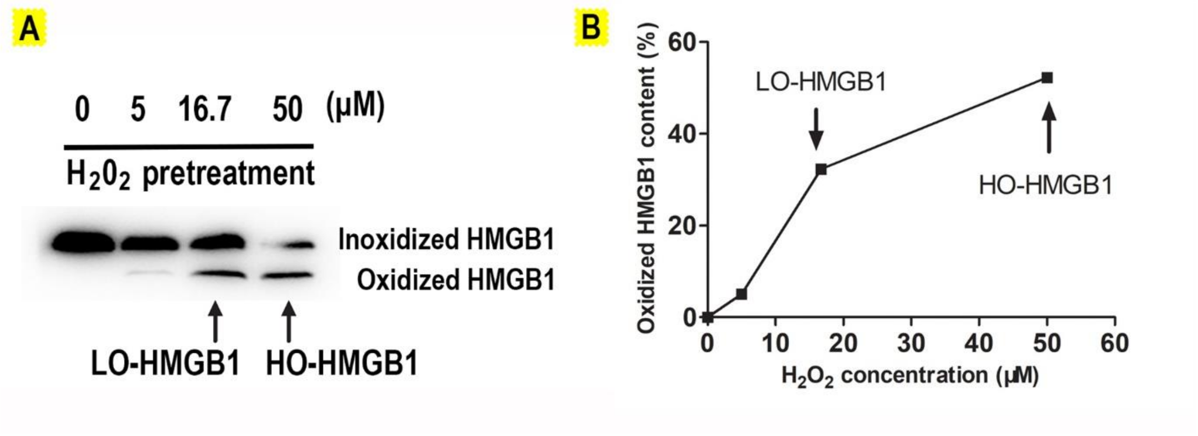

Figure S1: Determination of oxidized HMGB1 content in the mixtures of inoxidized and oxidized HMGB1. Oxidized HMGB1 was separated on SDS-PAGE gel and detected by Western blot with HMGB1 antibody (A). The mixtures with approximately 30% and 50% oxidized HMGB1 were labeled as LO-HMGB1 (low-level oxidized HMGB1) and HO-HMGB1 (high-level oxidized HMGB1), respectively (B).

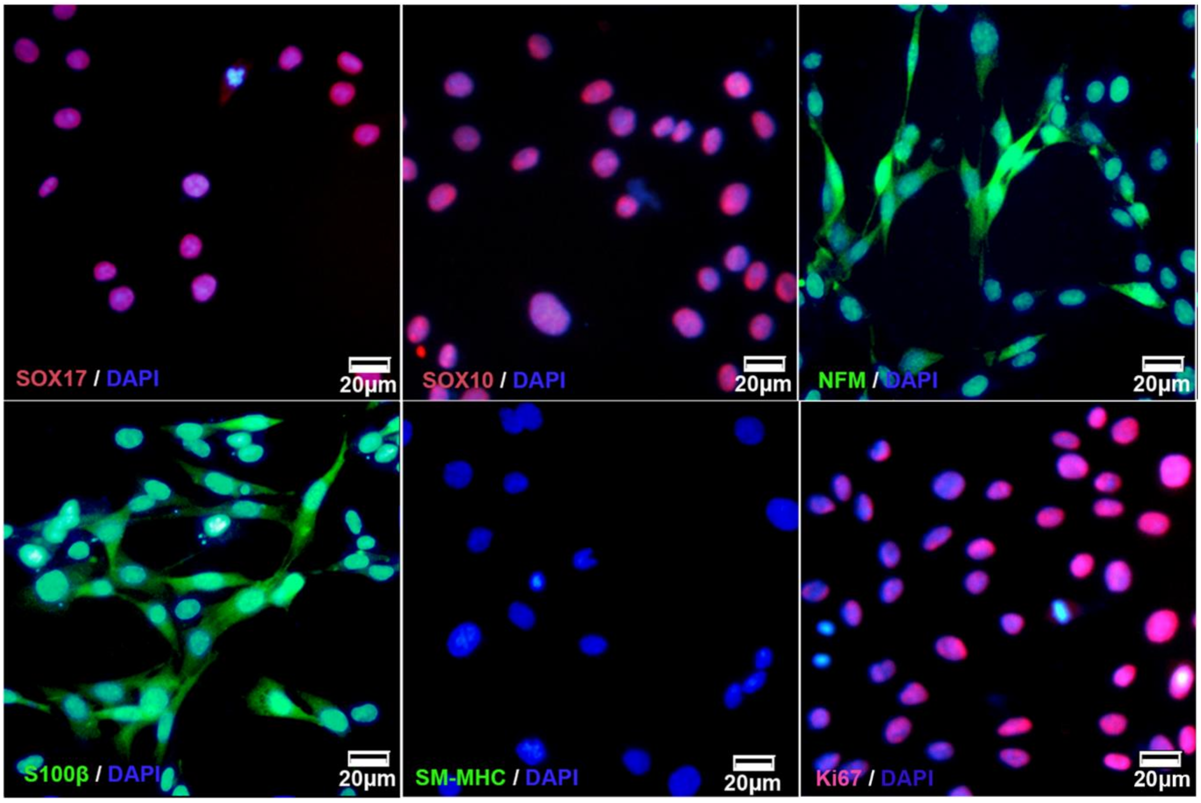

Figure S2: Immunofluorescent assay for MVSC markers on isolated cells. The cells were positive for Sox17, Sox 10, neural filament-medium polypeptide (NFM) and S100β while lacking smooth muscle myosine heavy chain (SM-MHC). Ki67 expression indicated that the cells were highly

proliferative.
